# Women and HIV

**DOI:** 10.1186/1472-6874-4-S1-S27

**Published:** 2004-08-25

**Authors:** Marene Gatali, Chris Archibald

**Affiliations:** 1 Centre for Infectious Disease Prevention and Control, Health Canada, Tunney's Pasture, Ottawa, Canada; 2 Centre for Infectious Disease Prevention and Control, Health Canada, Tunney's Pasture, Ottawa, Canada

## Abstract

**Health Issue:**

The epidemic of human immunodeficiency virus (HIV) and acquired immunodeficiency syndrome (AIDS) in developed countries has changed from the early epidemic that affected primarily men who have sex with men, to one that increasingly affects other groups such as injecting drug users (IDU) and heterosexuals. As a result, the number and percentage of women with HIV and AIDS is increasing.

**Key Findings:**

The number of women in Canada living with HIV, including those with AIDS, has increased over time. An estimated 6,800 women were living with HIV at the end of 1999, an increase of 48.0 % from the 1996 estimate of 4,600. On an annual basis, women account for a growing proportion of positive HIV test reports among adults in Canada. This proportion increased from 10.7% in the period 1985–95 to 25% in 2001. Heterosexual contact is the main risk factor for HIV infection in women, accounting for 63% of newly diagnosed cases of HIV infection in adult Canadian women in 2001; the majority of the remainder is due to IDU.

**Key Data Gaps and Recommendations:**

Research is needed to address specific information gaps regarding risk behaviours, testing patterns and HIV incidence and prevalence in women. This research needs to include the broader contextual factors that influence women's lives and their risk of HIV infection. Programmes and prevention efforts must be gender and age-specific and should target not only individual behaviours, but also the social and cultural context in which these behaviours occur.

## Background

The epidemic of HIV and AIDS in developed countries has changed from the early epidemic, which affected primarily men who have sex with men, to the current one, which increasingly affects other groups such as injecting drug users (IDUs) and heterosexuals. As a result of this shift, the number and percentage of women with HIV and AIDS is increasing. In addition, HIV infections in women have the potential for transmission to their infants.

Although HIV/AIDS affects both men and women, women are more vulnerable for biological, epidemiologic and social reasons. The broader situations and social conditions that fuel the HIV epidemic, such as marginalization, poverty and particularly gender power inequalities, increase women's vulnerability to HIV infection.

HIV manifests itself differently among women and men, especially with regard to early symptoms and later opportunistic infections. The presence of recurrent and persistent gynecological infections may be the first clinical manifestation of HIV in an infected woman and can occur early in the course of infection. Common early manifestations of HIV disease in women are recurrent or persistent vaginal candidiasis (yeast infections), menstrual changes, pelvic inflammatory disease, human papillomavirus infections, cervical abnormalities and cervical cancer[1,2].

The two principal ways that adult women acquire HIV are through injecting drug use and heterosexual contact with an HIV-infected partner or a partner who is at risk of HIV infection. Women who work as commercial sex workers, those who inject drugs, and partners of IDUs are at increased risk of acquiring HIV. Heterosexual contact is the main risk factor for HIV among women worldwide.

As of December 31, 2001, the World Health Organization (WHO) estimated that there were 17.6 million women living with HIV, constituting 47.3% of the estimated number of adults living with HIV infection in the world[1]. This percentage has risen dramatically from 20 years ago, when the AIDS epidemic was first detected. There is emerging new evidence of rising HIV infection rates among women in North America, parts of Europe and Australia. Unsafe sex and injecting drug use are propelling these epidemics, which at the same time are shifting towards more vulnerable communities. In many parts of the world, young disadvantaged women are being infected with HIV at rates higher than their male counterparts[1,3].

Recent estimates indicate that the number of women in Canada living with HIV, including those with AIDS, continues to grow. By the end of 1999, an estimated 6,800 women were living with HIV, an increase of 48% from the 1996 estimate of 4,600[4]. On an annual basis, women account for a growing proportion of positive HIV test reports with known age and sex among adults in Canada. This proportion increased from 10.7% in the years between 1985 and 1995, to 22% in 1998 and 25% in 2001[5].

This section reviews surveillance data and other epidemiologic publications to give an overview of HIV and AIDS in women in Canada. As well, the Canadian situation will be examined in the context of data from other developed countries.

## Methods

AIDS is a clinical diagnosis made by a physician. Cases are reported to provincial or territorial officials and then to the national level at the Centre for Infectious Disease Prevention and Control (CIDPC), Health Canada. HIV infection is a diagnosis made as a result of a positive laboratory test in an individual who requests an HIV test or is recommended to have one by his or her health care provider. The surveillance data presented here are derived from reported cases of AIDS and positive HIV tests across Canada. The databases MEDLINE, AIDSLINE and EMBASE were searched from 1996 onwards for relevant studies in English, and a scan of the WHO and UNAIDS Web sites was also done to obtain additional information on global HIV/AIDS cases and trends. In addition to these databases, relevant research studies and conference abstracts available at CIDPC were reviewed.

## Results

### Reported AIDS Cases

In Canada, of the 17,810 cumulative AIDS cases in adults reported to CIDPC up to December 31, 2001, there were 1,403 cases in adult women (7.8%)[5]. The proportion of women among adult AIDS cases with known age and sex has increased over time, from 5.6% before 1992 to 8.3% in 1995, peaking at 15.1% in 2001[5].

### Reported Positive HIV Tests

**Figure 1:**
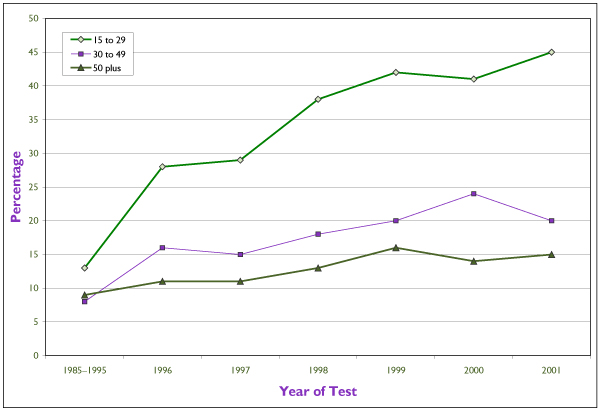
**Proportion of Women among Adult (≥ 15 Years) Positive HIV Test Reports, by Age Group and Year of Test (1985/95–2001) **Source: Data from HIV and AIDS in Canada, Surveillance Report to December 31, 2001.

Data from provincial and territorial HIV testing programs indicate that a total of 5,956 positive HIV tests with known age and gender were reported in adult women up to December 31, 2001. The proportion of HIV positive tests among females has risen over time, from 10.7% in the years between 1985 and 1995 to nearly 25% between January 1999 and December 31, 2001. This proportion varies considerably by age and is highest among adolescents and young adults. The proportion of females among positive HIV test reports in those aged 15 to 29 years has steadily increased from 14.6% between 1985 and 1995 to 44.5% in 2001[5]; trends among older women (30 years and older) show only slight increases over time (Figure 1). Note that proportions should always be examined in conjunction with the corresponding absolute numbers (Figure 5); the annual number of test reports among women aged 15 to 29 has gradually increased over time, whereas among men it has decreased.

Since the number of positive HIV tests is not a direct estimate of the annual number of new infections (see "Data Limitations"), CIDPC uses information from AIDS case reports, provincial/territorial HIV testing databases, population-based surveys, targeted epidemiologic studies and census data to estimate the true incidence. Since the 1980s, the estimated proportion of new HIV infections among women has increased. In 1999, of the estimated 4,190 new HIV infections, 920 (21.9%) occurred in women[4].

### Risk Factors

**Figure 2:**
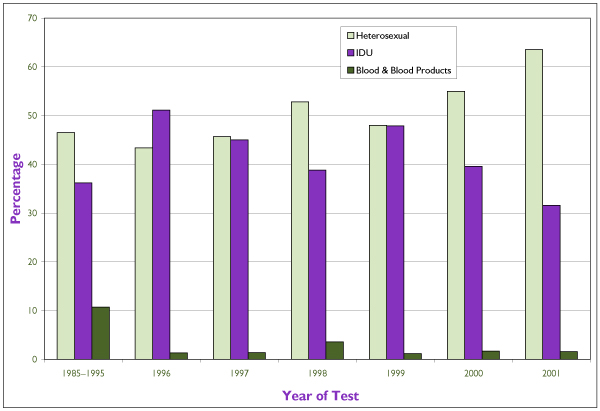
**Distribution of Exposure Category among Adult (≥ 15 Years) Female Positive HIV Test Reports, by Year of Test, Canada (1985/95–2001) **Source: Data from HIV and AIDS in Canada, Surveillance Report to December 31, 2001

The primary exposure categories for Canadian women are heterosexual contact and injecting drug use, which together accounted for 95.1% of newly diagnosed HIV infections in 2001, an increase from 82.7% in the period 1985–1995. Over time, there has been a slight increase in the proportion of positive HIV test reports attributed to heterosexual contact and a decrease in the proportion attributed to injecting drug use (Figure 2); these trends are also reflected in the absolute number of cases in the respective categories (Figure 6). Heterosexual contact is now the main risk factor for HIV infection in women, accounting for 63% of newly diagnosed cases of HIV infection in adult Canadian women in 2001[6].

### Heterosexual Contact

In Canadian HIV/AIDS surveillance data, the heterosexual contact category includes individuals who reported heterosexual contact as their most likely source of infection. This could be from sexual contact with a person who is either HIV infected or who is at increased risk of HIV infection – for example, an IDU, bisexual male or someone from an HIV-endemic country.

The percentage of women whose HIV status is attributed to heterosexual exposure has steadily increased, from 46.5% in 1985–1995 to 63.5% in 2001[5]. Of the reported heterosexual HIV cases, 44% are estimated to be due to sexual contact with an injecting drug user. Extrapolating to all new infections among women, an estimated 20% are related to heterosexual contact with an IDU partner, as compared with 5% of new infections in men. Therefore, non-IDU women who are sexual partners of male IDUs form a significant part of the Canadian HIV epidemic[7]. Heterosexual transmission of HIV most frequently occurs with repeated sexual contact with an HIV-infected person or people. However, there are other factors that might increase a woman's susceptibility to acquiring HIV infection, such as anal sex, sexually transmitted infections (e.g. gonorrhea, genital herpes, chlamydia), coexisting genital tract inflammation, ulcers or abrasion, traumatic sex (for example, vaginal bleeding during sex), contraceptive practices and sexual intercourse during menstruation[2,3].

Receptive anal intercourse has long been identified as a strong predictor of HIV transmission between male sexual partners. However, this sexual practice is rarely discussed as a primary factor for heterosexual transmission, despite studies demonstrating that anal intercourse carries a higher risk than vaginal intercourse[8].

Limited data suggest that HIV is less common among lesbians than heterosexual women[9,10].

### Injecting Drug Use

In Canada, there has been a steady increase in the proportion of adult AIDS cases attributed to IDUs, from 17.8% before 1996 to 26.6% in 1996 and 34.9% in 2000[5]. Three quarters of new HIV infections among women are directly or indirectly related to injecting drug use[7]. In addition to sexual exposure through IDU partners and the trading of sex for drugs or money, women may be exposed from their own drug use through the sharing of needles and other drug paraphernalia such as syringes, cotton balls and spoons. Of all these, the sharing of needles poses the greatest risk.

In a study in Ottawa, it was reported that 30% of female IDUs had shared needles in the previous six months[11]. Some studies have shown that the practice of sharing needles and other injecting behaviours in women are greatly affected by relationships with their regular male partners. Female IDUs are less likely than men to shoot up alone and are more likely than men to have an IDU sexual partner. Women more often than men reported sharing needles with their regular partner and being injected by their partner [12-14].

There have been reports of an elevated incidence of HIV among female as compared with male IDUs[15,16]. A recent study in Vancouver estimated the HIV incidence rate among female IDUs to be about 40% higher than that among male users[15]. An additional risk among female IDUs is the trading of unprotected sex for money and drugs. In the Vancouver injection drug use study, 437 of 1,293 participants were female, of whom 318 (73%) identified themselves as sex trade workers[16]. This study found that among IDUs who were sex trade workers in Vancouver during 1999–2000, the prevalence of HIV was 36.8%, whereas in Montreal in the same year the prevalence among IDUs who were sex trade workers was 10.5%[16,17].

Studies reveal that the mechanisms compromising women's injection and sexual safety are intricately and intimately connected to life histories characterized by emotional, sexual and physical abuse. For women, previous sexual or physical victimization may be a predisposing factor to drug use. It has been found that the prevalence of drug abuse or dependence was four times as high among women with a history of sexual assault as among other women[14].

### Vulnerable Subgroups

#### Young Women

**Figure 3:**
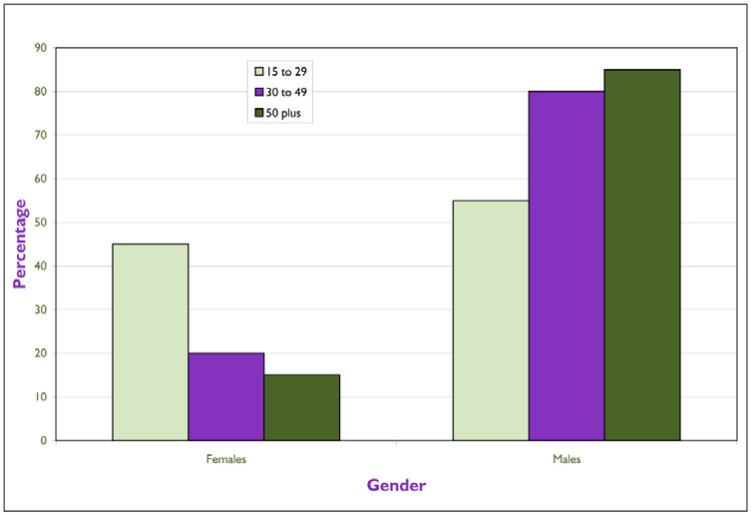
**Proportion of Women and Men among Positive HIV Test Reports, by Age Group in 2001 **Source: Data from HIV and AIDS in Canada, Surveillance Report to December 31, 2001.

The proportion of females among positive HIV test reports varies considerably by age and is highest among adolescents and young adults. Among positive HIV tests for all males and females in 2001 in Canada, females in the 15 to 29 year range accounted for 44.5% of all positive HIV test reports, an increase from 41% in 2000[5]. The proportion of adult males among reported HIV test reports is highest in the older age groups (Figure 3).

The limited data available about this group indicate that young women are involved in behaviours that put them at increased risk of contracting HIV infection. In the 1996–1997 NPHS, 25.6% of female participants in the 15 to 19 age group reported that they had had intercourse by the age of 15 years, as compared with 20% of males in the same age group. Among sexually experienced single respondents, 22% of females aged 15 to 24 reported that they had had more than one sexual partner in the previous 12 months[18].

Research suggests that many Canadian youth are having unprotected sexual intercourse. In the 1994–1995 NPHS, of sexually active individuals aged 15 to 19 years, 51% of females as compared with 29% of males reported never or only sometimes using a condom in the previous year. Among those aged 20 to 24, the corresponding percentages were 53% and 44%. The pregnancy rate in young women aged 15 to 19 years in Canada in 1997 was 42.7/1,000 and the abortion rate was 21.5/1,000 women. In 2000, the reported incidence of chlamydia and gonorrhea in Canada was highest among females aged 15 to 19 years: 1,236 and 96.4 per 100,000 women respectively[18]. In a study of Montréal street youth it was found that, of the sexually active participants, almost half the girls (47%) had been pregnant. It was also found that 41% of girls as compared with 22% of boys reported having had at least one sexually transmitted infection[19]. The differing rates of risky sexual behaviours and, as a result, of sexually transmitted diseases between young women and men can be attributed in part to the "power" within intimate relationships. The power in a relationship affects the woman's ability to take action to minimize acquiring HIV infection, such as negotiating condom use or the type of sexual activity she is involved in.

As a result of the synergy between HIV infection and other STD infection, the presence of other STDs increases the efficacy of transmission of HIV and increases the susceptibility to infection[2]. In addition to sexual risk, young women are at risk through injecting drug use. A 1998 study involving Winnipeg IDUs reported that HIV prevalence was higher among young women than young men aged 20 to 24: 12.1% versus 8.3%[20].

#### Women in Prison

Women in prison are at risk of HIV transmission as a result of injecting drug use as well as risky sexual behaviours. The practice of sharing needles and other injecting equipment can be exacerbated in prisons and jails because of the extremely limited availability of sterile injecting equipment. Two studies carried out in Quebec prisons reported a higher overall HIV prevalence rate among female than male inmates (9.8% versus 3.6% in one study and 7.6% versus 2.2% in the other) as well as higher rates among female than male IDUs (16% versus 7.7% and 15.6% versus 8.5%)[21,22]. As well, both studies found that HIV seropositivity among female inmates was related to prostitution and contact with an HIV positive partner, either sexually or through injecting drug use.

It is important to note that the higher rates of HIV infection among women reported here may include infections that were acquired before incarceration and not necessarily acquired in prison. The social conditions that put women at increased risk of incarceration include some of the factors that put them at increased risk for HIV infection.

#### Ethnic Groups

Relatively few HIV/AIDS studies in Canada have addressed ethnic background. Since the beginning of the AIDS epidemic in 1982, 85% of all AIDS case reports have included information on ethnicity[5,23]. Overall, Aboriginal and Black people are overrepresented among reported AIDS cases in Canada. The proportion of women among cumulative AIDS cases is higher among Aboriginal than non-Aboriginal cases (23.4% versus 8.0%); of the reported AIDS cases among Aboriginal women for whom the exposure was known, in 64.9% the risk factor was injecting drug use and in 30.9% it was heterosexual contact[5,24].

**Figure 4:**
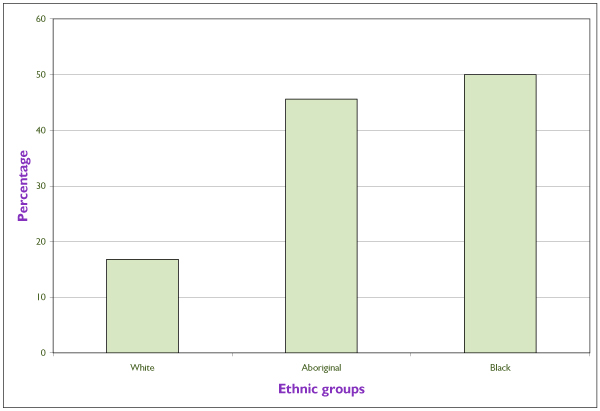
**Proportion of Women among Positive HIV Test Reports, by Ethnic Group, 1998–2001 **Source: Data from HIV and AIDS in Canada, Surveillance Report to December 31, 2001.

**Figure 5:**
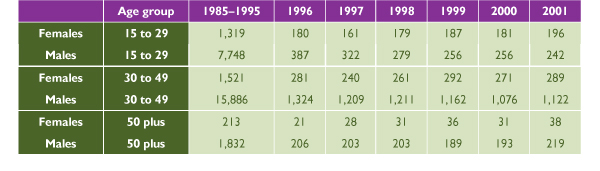
**Number of Positive HIV Test Reports among Adult Females and Males(≥ 15 Years), by Age Group and Year of Test, 1985–2001 **Source: Data from HIV and AIDS in Canada, Surveillance Report to December 31, 2001

**Figure 6:**
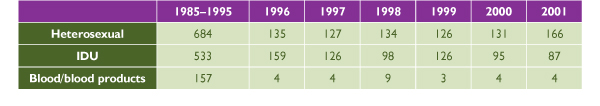
**Number of Positive HIV Test Reports among Adult Females (≥ 15 Years), by Year of Test and Exposure Category, 1985–2001 **Source: Data from HIV and AIDS in Canada, Surveillance Report to December 31, 2001

Ethnicity data for positive HIV test reports have been available only since 1998 and only for some provinces and territories. Approximately half of all HIV-positive test reports among Aboriginal people (45.6%) and Black people (50%) are from females as compared with 16% in the white population[23,24]. (Figure 4).

There are very few data on the prevalence and seroconversion rates of HIV among immigrant women or women from HIV-endemic countries. In one such study, the HIV prevalence rates among immigrant women were found to be slightly higher than the rates among Canadian-born women and women born in non-endemic countries[25]. Women from endemic countries are identified as being at increased risk of HIV because of their probable exposure through heterosexual contact and possible exposure to contaminated blood and blood products or medical equipment in the country of origin. In a study involving women undergoing abortion in Montréal from 1989 to June 2000, it was found that 85% of observed, positive test results were from women born in HIV-endemic countries. The HIV prevalence rate among women born in Haiti was 1.97% and among those born in HIV-endemic countries other than Haiti was 0.50%, whereas the rate among those born in Canada and other non-endemic countries was 0.06%[26]. In a similar study in 1989–1999, the risk factors identified for HIV infection included being Haitian-born, having had sex with a man at risk of HIV, having received a blood transfusion, and using injection drugs[27].

#### Pregnant Women

The rate of HIV infection among pregnant women is of great concern because of possible transmission to the unborn child. Over the past decade, the number of infants born to HIV-infected mothers (i.e. HIV-exposed infants) has increased from 56 in 1991 to 138 in 2001[5]. Of the reported total of 1,384 infants who were exposed to HIV from their mothers between 1984 and 2001, 375 have been confirmed to be HIV positive, and an additional 56 have indeterminate serostatus and are currently being monitored (data from the Canadian Pediatric AIDS Research Group)[5].

In all Canadian provinces, HIV testing of pregnant women remains the choice of the woman. Guidelines and recommendations have been adopted to encourage the provision of HIV tests with informed consent as part of the routine prenatal tests. There is now evidence that mother-to-child transmission can be greatly reduced by antiretroviral treatment[28,29]. The HIV transmission rates to newborns without treatment is estimated at approximately 24%. This rate can be reduced by three quarters or more with treatment[30]. A study looking at trends of antiretroviral drug use in Canada found that the perinatal HIV transmission rate was reduced to 4.8% with zidovudine monotherapy and to 2.5% with combined therapy[28,29].

The proportion of pregnant women known to be HIV positive who receive antiretroviral therapy in Canada increased from 37% in 1994 to 84% in 1998[29]. An analysis to estimate the number of HIV infections among infants that could be prevented annually in Canada by an active antenatal HIV testing program found that an estimated 36 HIV infections (65% reduction) could be prevented among infants each year in Canada by a program of 90% testing compared with no testing[30].

In pediatric centres across Canada where children and HIV-infected mothers were followed between 1995 and 1997, 19% (*n *= 259) were Aboriginal women. Despite the high numbers of Aboriginal women seen at HIV clinics and pediatric centres, the data show that these women are as likely to be taking antiretroviral therapy (62%) as pregnant Black (63%) and Caucasian women (66%). However, 38% of Aboriginal and 27% of Black women receive antiretroviral therapy late in their pregnancy (in the third trimester or intrapartum) as compared with 9% of white women[24].

Studies of pregnant women can provide an important source of information on the prevalence rate of HIV in the general heterosexual population. Prenatal seroprevalence studies in Canada report an estimated national rate of HIV infection among pregnant women of approximately 3 to 4 per 10,000 population[6,28]. Large metropolitan areas have the highest rates: for example, 4.7 per 10,000 in Vancouver versus 3.4 in the rest of British Columbia in 1994[6], and 15.3 in Montréal versus 5.2 in the rest of the province of Quebec in 1990[31]. However, even provinces without large metropolitan areas have significant rates; for example, 4.1/10,000 in New Brunswick during 1994–1996[32].

### HIV Testing

HIV testing patterns within the general population, along with the profile of the people being tested, are important for designing and targeting intervention programs as well as developing a context for HIV/AIDS surveillance data. There are currently three HIV testing options available in Canada, depending on the province or territory: anonymous, nominal/name based and non-nominal/non-identifying HIV testing[33]. The availability of multiple testing options increases the chances of women being tested. People with risk factors for HIV are more likely to have been tested for HIV than those without such risk factors.

If HIV is diagnosed late (long after infection and close to the time of AIDS development), then the individual is unaware of his or her HIV status for a long time and, in addition, along with his or her sexual and/or IDU contacts cannot benefit from the new HIV treatment and appropriate counselling to reduce potential risk behaviours. In Canadian AIDS surveillance data, approximately half of both males (50%) and females (54%) test late for HIV (HIV diagnosed less than 12 months before AIDS diagnosis). Those who were Canadian born were less likely to test late (46%) than those born outside Canada (65%). Late testing was significantly more common in Aboriginal, Asian and Black communities (75.0%, 68.2% and 68.2% respectively) than among whites (45.2%). In female AIDS cases, transmission through heterosexual contact, having no identified risk and being non-white were predictors of testing late for HIV infection[34].

### International Data

The rest of the developed world is experiencing a similar trend of increasing HIV infections among women. An overlap of injecting drug use and heterosexual intercourse appears to be driving the HIV epidemic among women[1]. In eastern Europe, however, HIV infections are predominantly due to injecting drug use[35].

In the United States, 40,585 HIV cases in adult and adolescent females were reported up to June 2001, accounting for 39% of all adult and adolescent HIV cases[36]. African-American women have the highest AIDS rate of any race or ethnic group of women in the United States, and they account for 67% of female HIV cases[36,37]. A Los Angeles study found that these women tended to be unemployed (88%), single mothers (64%), living on public assistance (86%), with annual household incomes of less than US$10,000 (76%), and with a history of crack use (50%). Compared with women of other races with HIV and AIDS, they reported more sexual partners (61%), more sexually transmitted infections, sought treatment for their HIV infection later, and were more likely to trade sex for drugs or money[38].

In the United Kingdom and some parts of Europe and Australia, the increasing number of HIV infections in women is mainly due to heterosexual contact in HIV-endemic countries or with partners from HIV-endemic countries. Migrants from endemic countries account for the largest proportion of HIV positive women in these countries[35,38,39]. In the U.K., more than three quarters of HIV infections resulting from heterosexual exposure (9,077 of 11,555) are recorded as having been acquired abroad, primarily in Africa. With the rise in the number of those who acquired their HIV infections heterosexually, there has been an increase in the U.K. in the number of diagnoses in women. The male-female ratio for all new infections diagnosed in 1985–1986 was approximately 14:1, whereas in 1998–1999 it was about 2.5:1[38,40].

In a study looking at the ethnic differences among women infected with HIV in Britain and Ireland, researchers found that Black women had fewer sexual partners (3) than their white counterparts (7), and that the main route of transmission was heterosexual contact for Black women (93%) and a combination of heterosexual contact (43%) and injecting drug use (55%) for white women. They also found that for Black women, the infection was diagnosed at a much later stage[41]. These findings warrant further research into the differences that might exist between ethnic groups in order to plan appropriate interventions.

## Discussion

Despite their limitations, the available data show an increase in the proportion of women among adult HIV test reports over time. This proportion varies with age and is highest in the 15 to 29 age group among both HIV test reports and reported AIDS diagnoses in Canada.

This chapter focused on the epidemiologic factors that put women at increased risk of HIV infection. However, these factors are interconnected with social factors in women's lives. Available research indicates that women's risk of acquiring HIV infection occurs within the context of their interpersonal relationships. This social context is characterized by the HIV risk behaviours of the community of people the woman belongs to, the cultural norms that prescribe her behaviour within sexual relationships, and her personal power within her intimate relationships. These factors affect the woman's ability to take action to minimize acquiring HIV infection, such as negotiating condom use or the type of sexual activity she is involved in [42-44].

HIV appears to be a particular problem for certain subgroups of women: women in prison, Aboriginal women and Black women. The isolation and marginalization in these ethnocultural communities because of economic and social circumstances, language barriers, access to resources and cultural expectations are likely risk factors for HIV transmission[43,44].

### Data Limitations

Changes in the number of reported HIV positive tests as well as the proportions and trends reported in this chapter must be interpreted with caution. Surveillance data from positive HIV test reports are not direct estimates of the annual number of new HIV infections. This is because only a proportion of those newly infected in a given year are tested for HIV in that same year. Furthermore, not all new HIV test reports in a given year are due to HIV infections that occurred in that year, since many individuals will have been infected several years before their first HIV positive test[5]. In addition, data on risk behaviours, especially among young women, are not taken from recent surveys and may be outdated.

### Implications and Recommendations

HIV in women encompasses many facets of women's lives and behaviours. Reducing the toll of the HIV epidemic among women will require efforts on several fronts:

• There needs to be more research on risk behaviours and HIV incidence and prevalence among women, especially in particular subgroups, such as women in prison, young women, Aboriginal women and those from endemic countries. This research needs to include the influence of the broader contextual factors on women's lives and on their risk of HIV infection.

• More research on HIV testing behaviours and patterns among women is needed. This will help the individuals to receive appropriate counselling and treatment as well as help public health officials to better interpret surveillance data.

• Programs and prevention messages need to be tailored to specific social, cultural and economic contexts that may make women more vulnerable to HIV infection than men.

• Prevention efforts must be sex- and age-specific and should target not only individual behaviours but also the social and cultural context in which these behaviours occur (for example, increasing individual motivation to use condoms and reducing contextual barriers to their use).

• Specific interventions are needed to address the intersection of injecting drug use and HIV transmission. These interventions should target both women who inject and those who do not inject but have sexual partners who do. For both groups of women, violence, unequal gender roles and power differentials are important elements that relate to HIV risk behaviour.

• There needs to be an increased emphasis on appropriate HIV prevention, counselling and testing as well as treatment services for women.

## Note

The views expressed in this report do not necessarily represent the views of the Canadian Population Health Initiative, the Canadian Institute for Health Information or Health Canada.
